# Self-selection in a randomized trial of web-based primary and secondary prevention alcohol brief intervention

**DOI:** 10.1186/1940-0640-8-S1-A10

**Published:** 2013-09-04

**Authors:** Nicolas Bertholet, Joseph Studer, John A Cunningham, Jean-Bernard Daeppen, Gerhard Gmel, Bernard Burnand

**Affiliations:** 1 Alcohol Treatment Center, Department of Community Medicine and Health, Lausanne University Hospital, Switzerland; 2 Centre for Mental Health Research, Australian National University, Australia; 3 Clinical Epidemiology Center, Department of Community Medicine and Health, Lausanne University Hospital, Switzerland

## 

How much a randomized controlled trial (RCT) sample is representative/differs from its declared source population is a challenging question, with major implications with regard to generalizibility of results. The question is crucial for freely available web-based interventions tested in RCTs.

We compared participants in a primary/secondary prevention web-based alcohol brief intervention RCT to its source population. There is a mandatory army recruitment process in Switzerland at age 19 for men. Between 8.2010 and 7.2011, 12,564 men attended two recruitment centers (source population) and were asked to answer a screening questionnaire on substance use; of the 11,819 (94%) who completed it, 7,034 (56%) agreed to participate in a cohort with regular assessments. In 2012, irrespective of their drinking, cohort participants were invited to the RCT; 1,549 agreed to participate. Using chi-square and t-test, we compared screening data of RCT participants to other members of the source population with respect to weekly alcohol use, maximum number of drinks/occasion, Alcohol Use Disorders Identification Test (AUDIT), prevalence of binge drinking (≥6 drinks per occasion at least monthly), unhealthy alcohol use (>210g of ethanol/week and/or ≥1 binge episode/month), and abstinence.

RCT participants (n=1,549) drank less than other members of the source population (n=10,270): they reported a mean (SD) of 6.2(8.1) vs 7.4(10.7) drinks/week, t=5.2, p<.0001; 9.7(7.7) vs 10.3(9.1) maximum number of drinks/occasion, t=2.6, p=.009; and 6.6(4.4) vs 7.2(4.9) AUDIT scores, t=4.7, p<.0001. The prevalence of binge drinking was lower among RCT participants (36.7% vs 43.9%, χ^2^=28.8, p<.0001), as was unhealthy alcohol use (37.1% vs 44.1%, χ^2^=27.5, p<.0001) and abstinence (7.8% vs 9.7%, χ^2^=5.8, p=0.016). RCT participants differed from other members of the source population: they reported less drinking and lower levels of consequences or risk, but were also less likely to be abstainers, indicating that the self-selection applies to both ends of the drinking spectrum.

